# Drivers and conservation impacts of innovative tree nesting in an isolated island population of the red‐billed chough

**DOI:** 10.1002/ecy.70344

**Published:** 2026-03-16

**Authors:** Guillermo Blanco, David Notario, Iñigo Palacios‐Martínez, Óscar Frías, Raymundo Montoya Ayala, Martina Carrete

**Affiliations:** ^1^ Department of Evolutionary Ecology Museo Nacional de Ciencias Naturales, CSIC Madrid Spain; ^2^ Facultad de Estudios Superiores Iztacala UNAM. UBIPRO Laboratorio de SIG y Análisis Espacial Tlalnepantla Estado de México México; ^3^ Department of Physical, Chemical and Natural Systems Universidad Pablo de Olavide Sevilla Spain

**Keywords:** Canary Islands, nesting innovation, palm tree, *Pyrrhocorax pyrrhocorax*

Innovativeness, defined as the ability to develop flexible behavioral responses to ecological challenges, plays a crucial role in enabling animals to cope with novel, changing, or restrictive environmental conditions (Reader & Laland, 2003). On oceanic islands, environmental features derived from the island's isolation, location and its geological setting, as well as the resultant “insular” biotic communities (Whittaker & Fernández‐Palacios, 2007), may promote behavioral innovations as a mechanism for niche shifts, fostering local adaptations and evolutionary divergence (Gavriilidi et al., 2022). Nesting innovations, in particular, may reflect plastic capacities to solve problems related to habitat features, predation pressure, nest‐site availability, foraging opportunities, and social constraints (Gavriilidi et al., 2022; Hernández‐Brito et al., 2022; Peluc et al., 2008). These innovations can have significant implications for the demography and conservation of isolated populations and endemic species (Covas, 2012; Whittaker & Fernández‐Palacios, 2007) through a breadth of life history traits such as growth rate and body size (Covas, 2012; Sandvig et al., 2019), survival (Beauchamp, 2021), and number of offspring (Jezierski, 2024). Behavioral innovations may also be related to plastic capacities due to changes in brain size on islands (Sayol et al., 2018).

The remarkable cognitive capacity of corvids, associated with their large relative brain sizes, is widely recognized as a key factor facilitating the emergence of behavioral innovations, allowing them to adapt flexibly to diverse ecological challenges and exploit novel opportunities (Taylor, 2014). The red‐billed chough (*Pyrrhocorax pyrrhocorax*), a medium‐sized corvid of the Western Palearctic, remains relatively understudied due to its rarity, elusive behavior, and preference for remote and inaccessible environments. It is considered an open‐habitat specialist, typically occupying steppes, pseudo‐steppe dry farmlands, and montane or coastal grasslands (Bignal et al., 1996; Blanco, Tella, & Torre, 1998; Morinha et al., 2017), and a diet‐specialist feeding predominantly on soil invertebrates (Kerbiriou & Julliard, 2007; Laiolo & Rolando, 1999; Sánchez‐Alonso et al., 1996). Across its range, the species is almost exclusively associated with rocky habitats and displays troglodytic nesting and roosting behavior, using cavities, crevices, caves, and chasms (Blanco et al., 2021). Recently, a nesting innovation involving the use of artificial structures that mimic the microclimate, concealment, and light conditions of natural cavities, such as abandoned buildings, mines, bridges, and wells, has been documented, facilitating range expansion into regions lacking natural rocky structures (Bignal & Bignal, 1987; Blanco et al., 1997; Саая, 2025).

A geographically isolated population of red‐billed choughs (hereafter, choughs) persists on the oceanic island of La Palma, in the Canary Islands (Spain), representing the southwestern‐most limit of the species' global distribution. This insular population exhibits the highest recorded breeding density and proportion of non‐breeding individuals (Blanco et al., 2009). Genetic studies suggest that it likely originated from a now‐extinct coastal lineage in North Africa, showing closer genetic affinity to Iberian populations than to nearby Moroccan continental populations and demonstrating both phenotypic and genetic divergence from mainland conspecifics (Morinha et al., 2020; Recuerda et al., 2024). In contrast to other populations, choughs in La Palma frequently forage in vegetated environments, including forests of endemic Canary Island pine (*Pinus canariensis*), humid montane woodlands and shrub‐dominated mid‐altitude zones with dense plant cover. There, they perch and forage on and beneath trees and shrubs, consuming fruits and invertebrates (Blanco et al., 2014; Pais & García, 2000). These foraging innovations may be linked to the historical absence of terrestrial predators and to the unique ecological conditions of the insular environment (Blanco et al., 2014; Gavriilidi et al., 2022). Islands often also foster behavioral innovations related to breeding due to reduced predator communities, low interspecific competition, patchy distribution of suitable nesting sites, limited availability of familiar food types, and distinct climatic conditions, among other factors (Blanco et al., 2014; Gavriilidi et al., 2022; Jezierski et al., 2024; Tebbich et al., 2010). The lack of native terrestrial predators, uneven availability of cliffs, and high habitat heterogeneity in La Palma could also encourage choughs' exploratory nesting behavior. In fact, nesting in buildings, Canary Island date palms (*Phoenix canariensis*), and Canary pines has been previously noted anecdotally in this population (Blanco et al., 2009; Ludwigs, 2004). The chough and the endemic Canary Island palm are iconic species of La Palma, serving as living symbols of the island's landscape, history, and biodiversity. Their interactions exemplify the deep biocultural links between wildlife and human heritage, highlighting how traditional landscapes have shaped, and been shaped by, these species.

Between March and April 2025, we conducted a comprehensive island‐wide census of breeding pairs following standard methods (Blanco et al., 2009). Briefly, active nests were located visually using binoculars and telescopes on cliffs, ravines, and caves, as well as in buildings and palms, with repeated visits to the largest ravines and cliffs, and whenever possible, by more than one observer. Direct evidence of breeding included territorial occupation of cavities, egg‐laying and incubation (with the male feeding the female), or nestling feeding. We identified 323 active breeding pairs. The majority (88.9%, *n* = 287) nested in deep ravines, large cliffs, and caves, both inland and along the coast. A smaller fraction (6.2%, *n* = 20) used anthropogenic structures such as buildings, bridges, tunnels, artificial walls, and other constructions. Notably, 16 pairs (5.0%) were found nesting in Canary Island date palms (hereafter, palm trees). Palm nests were primarily concentrated in the eastern and central parts of the island, across urban zones, agricultural landscapes and, occasionally, edges of pine forests and scrublands (Figure 1). These sites were generally found in areas with limited or no vertical rocky substrates, though some were near cliffs also occupied by nesting choughs. Nesting palms ranged from 5 to 12 m in height and were typically isolated or in small clusters. Nests were placed high on the trunk, tucked among old frond bases or within concavities sheltered by leaves, providing concealment and support (Figure 1). At least five additional palms known to host nests in previous years (based on reports from local residents and our observations since 2003) were unoccupied in 2025. Remarkably, one of these palm trees had supported nesting regularly since at least 2007 (own observations). Of the 16 palm‐nesting pairs, individuals were observed across various reproductive stages: nest building (14.3%, *n* = 2), incubation (35.7%, *n* = 5), and chick feeding (50.0%, *n* = 7). Two pairs' status could not be determined. Like individuals nesting on man‐made structures, choughs using palm trees appear to initiate breeding earlier than cliff‐nesting pairs (ordinal regression model: reproductive status [nest building, incubation, chick rearing] ~ substrate [palm trees/buildings or cliffs]; estimate for palm trees/buildings = 0.72, SE = 0.38, *t* = 1.88, *p* = 0.0597; fitted using the polr package in R). Additionally, we observed one territorial pair in a pine forest devoid of rocky formations. Although this site contains “witches' brooms”—dense pine branch clusters—previously used for nesting (Figure 1), as confirmed by local rangers, no active nests in these structures were located during the 2025 breeding season. A pair was observed nesting in an introduced palm (*Washingtonia robusta*) in March 2026.

**FIGURE 1:**
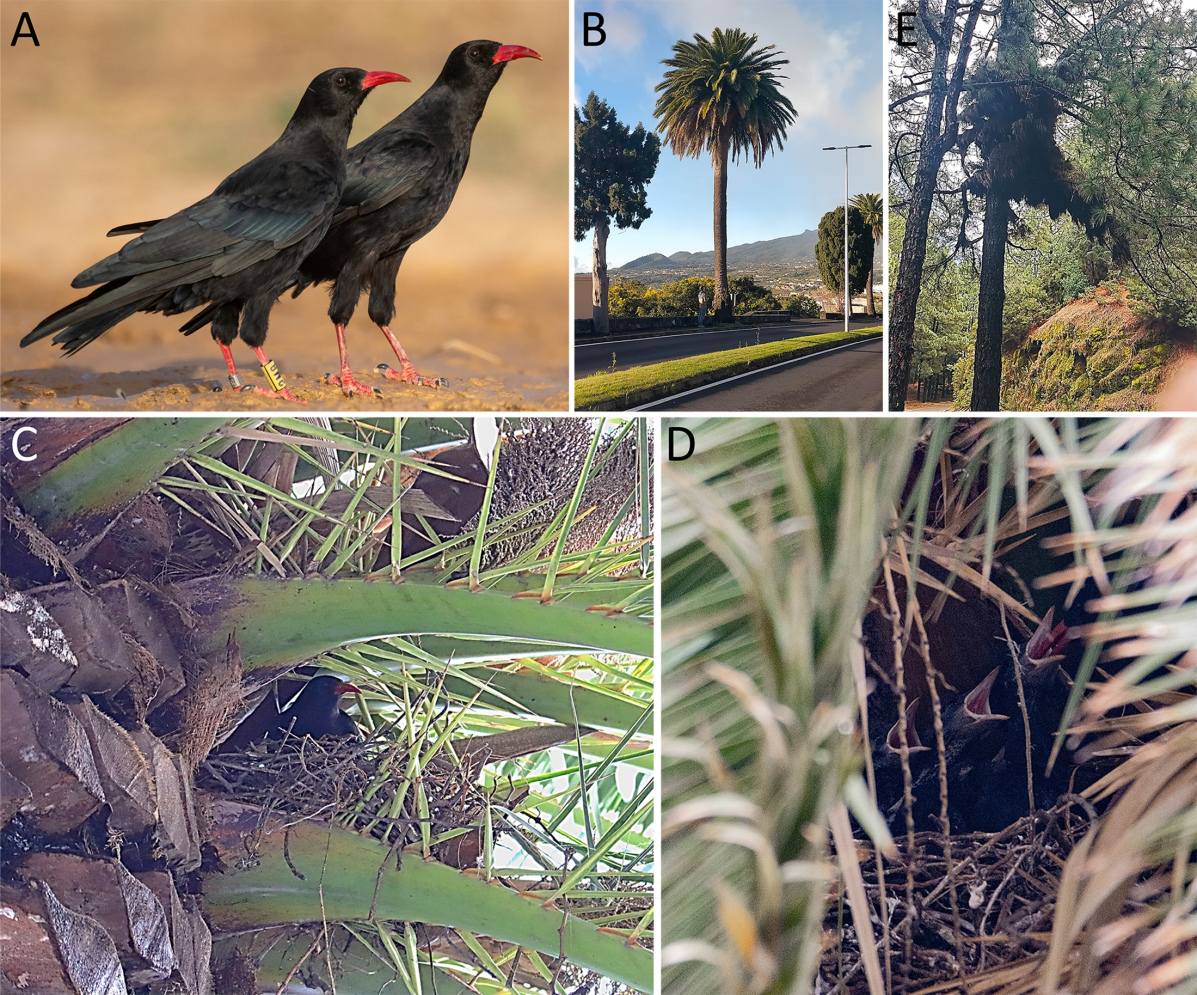
In La Palma, Canary Islands, red‐billed choughs, *Pyrrhocorax pyrrhocorax* (A), a medium‐sized corvid species, have started to display an innovative nesting strategy. Using Canary Island date palm trees (*Phoenix canariensis*), mostly located in urban settings (B), choughs build their nests tucked among old frond bases (C: female incubating; D: adult feeding nestlings). Choughs in La Palma also use witches' broom structures (dense branch clusters with cavities) in Canary Island pines (*Pinus canariensis*) (E). Photo credits: (A) Arnau Guardia; (B) and (E) Guillermo Blanco; (C) and (D) David Notario.

The use of palm trees as a nesting substrate by choughs in La Palma represents a striking behavioral innovation in a species known for its strong association with rocky environments. Across most of its range, the red‐billed choughs largely avoid trees, even for perching. Most palm nests were located in areas lacking cliffs or caves but offering nearby foraging habitats, such as agricultural mosaics, open urban edges, and pine forest margins. In this context, palm trees, together with man‐made structures, may serve as the only available nesting substrates. As with populations that use artificial substrates (Blanco et al., 1997), palm nesting appears driven by a need to expand available breeding space. This can reduce competition, allow pairs to nest closer to foraging areas, and lower energy costs and exposure to potential threats (Banda & Blanco, 2009).

Local reports and our observations indicate that palm nesting has occurred sporadically over the past two decades. However, while no complete census had been conducted previously, our finding suggests a recent increase in both frequency and spatial extent. Many currently occupied palm trees were certainly unused in past years, based on the fact that these sites have been frequently visited by us over the past two decades without any sign of occupation. This absence could not have been overlooked given the readily observable behavior and the marked tameness of the species on the island, pointing to a recent increase in palm use. Most palm trees are located in inhabited areas, typically near single‐family homes with gardens and small‐scale agricultural plots dispersed across the island. In fact, the majority of palm trees on the island are planted (Sosa et al., 2021). Therefore, this behavioral shift may have been unintentionally favored by human‐driven landscape modifications.

Foraging of choughs on palm trees for invertebrates (own observations) may have increased their familiarity with these structures, lowering the threshold for their use as nesting sites. This adaptation has likely been further facilitated by the near cessation of past human persecution, particularly the traditional practice of removing chicks from nests to keep as pets. The possibility that this behavior reflects more than individual‐level exploration is supported by repeated use of certain palms in consecutive years, suggesting site fidelity typical of this species (Banda & Blanco, 2014), and potential for social learning and cultural transmission (Aplin et al., 2025). Such transmission could facilitate the spread of this innovation across the area occupied by palm trees in the island, particularly if associated with successful breeding outcomes. Nesting in palm trees may confer reproductive advantages, such as enabling earlier breeding phenology. In this species, early‐season breeding is generally associated with larger clutch size and breeding success (Banda & Blanco, 2009; Blanco, Fargallo, et al., 1998), a pattern consistent across bird species (Newton, 1998). This benefit could arise from access to foraging areas near palms that are distant from the cliff zones where high‐density aggregations of breeding pairs occur, thereby reducing intraspecific competition. Field observations show that palm‐nesting pairs are distributed across different breeding stages, with about half already provisioning nestlings. If early breeders on palms achieve greater reproductive success due to advanced breeding, the resulting fitness benefits could promote the persistence and expansion of this strategy. Sustained over generations, it might represent a stable behavioral adaptation to local conditions, reinforcing the population's resilience to environmental change or habitat loss.

The significance of this behavioral novelty is amplified by its implications for territoriality and breeding system structure. Palm nests appear to be used by single territorial pairs rather than communal aggregations. This contrasts with cliff sites, which often host dense aggregations (up to 30 pairs) due to abundant cavities and spatial tolerance among breeders and floaters (Blanco et al., 2009). Palm nesting may enforce exclusive use of the surrounding area and may reflect an adaptive response to habitat saturation. In some cases, where palm trees were located very close to large cliffs that also hosted breeding pairs, their selection may reflect an active preference for this novel substrate. This could be related to microhabitat advantages or differences in travel costs to foraging areas, which can significantly reduce overall energy expenditure and intraspecific competition (Kerbiriou et al., 2006; McNab, 2002; Smith et al., 2025). Similarly, nesting in pine witches' brooms opens up the possibility of exploiting extensive pinewood foraging areas that lack rock‐based nesting sites, although this strategy appears more limited by the structural suitability and durability of these formations.

The relevance of palm nesting is heightened under conditions of habitat saturation, as it provides a release from density‐dependent constraints on nesting opportunities (Blanco et al., 2009, 2014). In particular, the unusually high proportion of non‐reproductive individuals in La Palma (Blanco et al., 2009) appears to result from individuals' difficulties in achieving reproductive status due to nutritional limitation of proteins (Blanco et al., 2014). Moreover, a “fence effect” associated with island isolation makes long‐distance escape dispersal to other islands in the archipelago or continental areas highly unlikely (Martín & Lorenzo, 2001; Morinha et al., 2020). This may increase survival rates and further elevate population density, intensifying intraspecific competition for reproductive resources rather than for basic maintenance (Blanco et al., 2014). Nesting innovations (nests in palm tree, pines, and man‐made structures) expand La Palma choughs' breeding habitat into previously unsuitable areas and may improve reproductive success under high‐density conditions. Therefore, this innovation may contribute to an overall increase in spatial distribution and size of the breeding population.

From a functional perspective, palm nesting partially decouples nesting and foraging requirements, increasing ecological plasticity. It may enable colonization of new areas and buffer against the natural transformation of areas due to volcanic activity (Blanco et al., 2024) or anthropogenic disturbance (Blanco et al., 2007; Pais, 2005). However, palm nesting also introduces new vulnerabilities. Unlike rock cavities, palm nests are exposed to meteorological threats and human disturbances. During this study, a hurricane‐level storm toppled a nesting palm tree, resulting in reproductive failure. Frond pruning also poses a risk, as it removes both structural support and cover, further compromising the suitability of these trees as breeding sites.

In conclusion, palm nesting by choughs in La Palma illustrates how ecological constraints can catalyze behavioral innovations. This shift challenges assumptions about the species' ecological niche and underscores its capacity for adaptation under changing conditions. While it offers potential demographic and conservation benefits, it also introduces new risks that merit attention. Continued monitoring and research will help determine whether this behavioral innovation represents a stable strategy or a short‐term, compensatory response to local limitations. Either way, it provides a compelling example of how island populations can diverge behaviorally from their continental counterparts, as well as an opportunity for conservation outreach involving two iconic species of the Canary Islands' cultural landscape.

## CONFLICT OF INTEREST STATEMENT

The authors declare no conflicts of interest.
